# Green Tea Polyphenol EGCG Sensing Motif on the 67-kDa Laminin Receptor

**DOI:** 10.1371/journal.pone.0037942

**Published:** 2012-05-29

**Authors:** Yoshinori Fujimura, Mami Sumida, Kaori Sugihara, Shuntaro Tsukamoto, Koji Yamada, Hirofumi Tachibana

**Affiliations:** 1 Innovation Center for Medical Redox Navigation, Kyushu University, Fukuoka, Japan; 2 Department of Bioscience and Biotechnology, Faculty of Agriculture, Fukuoka, Japan; 3 Bio-Architecture Center, Kyushu University, Fukuoka, Japan; University of Helsinki, Finland

## Abstract

**Background:**

We previously identified the 67-kDa laminin receptor (67LR) as the cell-surface receptor conferring the major green tea polyphenol (–)-epigallocatechin-3-*O*-gallate (EGCG) responsiveness to cancer cells. However, the underlying mechanism for interaction between EGCG and 67LR remains unclear. In this study, we investigated the possible role of EGCG-67LR interaction responsible for its bioactivities.

**Methodology/Principal Findings:**

We synthesized various peptides deduced from the extracellular domain corresponding to the 102-295 region of human 67LR encoding a 295-amino acid. The neutralizing activity of these peptides toward EGCG cell-surface binding and inhibition of cancer cell growth were assayed. Both activities were inhibited by a peptide containing the 10-amino acid residues, IPCNNKGAHS, corresponding to residues 161-170. Furthermore, mass spectrometric analysis revealed the formation of a EGCG-LR161-170 peptide complex. A study of the amino acid deletion/replacement of the peptide LR161-170 indicated that the 10-amino acid length and two basic amino acids, K^166^ and H^169^, have a critical role in neutralizing EGCG’s activities. Moreover, neutralizing activity against the anti-proliferation action of EGCG was observed in a recombinant protein of the extracellular domain of 67LR, and this effect was abrogated by a deletion of residues 161-170. These findings support that the 10 amino-acid sequence, IPCNNKGAHS, might be the functional domain responsible for the anti-cancer activity of EGCG.

**Conclusions/Significance:**

Overall, our results highlight the nature of the EGCG-67LR interaction and provide novel structural insights into the understanding of 67LR-mediated functions of EGCG, and could aid in the development of potential anti-cancer compounds for chemopreventive or therapeutic uses that can mimic EGCG-67LR interactions.

## Introduction

Green tea has been shown to have cancer preventive activity in a variety of organ sites in animal models [1]–[3] and humans [4]. Among the green tea constituents, (–)-epigallocatechin-3-*O*-gallate (EGCG) is the most abundant and most active constituent in inhibiting experimental carcinogenesis and related reactions. Although many mechanisms for the anti-cancer activities of EGCG at concentrations of more than 10 µM, concentrations that are much higher than those observed in plasma or tissues, have been proposed based mainly on studies in cell lines, it is still not clear which EGCG-induced molecular events and potential molecular targets are responsible for its cancer-preventive activity *in vivo* [5]. Recently we have identified the 67-kDa laminin receptor (67LR) as a cell-surface EGCG receptor that mediates the anti-cancer action, cancer cell growth inhibition, of a physiologically relevant EGCG [6], and others showed that RNAi-mediated silencing of 67LR results in abrogation of EGCG-induced apoptosis in myeloma cells [7].

The 67LR is a non-integrin cell-surface receptor for laminin with high affinity [8]. This receptor appears to be very peculiar since so far only a full-length gene encoding a 37-kDa precursor protein of 295-amino acids has been isolated and the mechanism by which the precursor reaches the mature 67-kDa form still remains unclear [9]. A hydrophobic segment (residues 86–101) has been hypothesized to act as a transmembrane domain, and the C-terminal domain of the receptor is thought to be involved in the interaction with laminin [10]. The evolutionary analysis of the sequence identified as the laminin-binding site in the human protein suggested that the acquisition of the laminin-binding capability is linked to the 173–178 region of the extracellular domain (residues 102–295), which appeared during evolution concomitantly with laminin [11]. Its role as a laminin receptor makes it an important molecule in cell adhesion to the basement membrane and in the metastasis of tumor cells [12]. Downregulation or inhibition of 37-kDa/67-kDa laminin receptor impedes invasion of fibrosarcoma cells, suggesting that the receptor plays a key role in tumor metastasis [13]. An increase in 67LR expression as compared with the corresponding normal tissue has been found in a variety of common cancers, including breast, cervical, colon, lung, ovary, pancreatic, and prostate carcinomas [14], [15]. Interestingly, the 67LR is the molecule showing a good correlation between the expression pattern of 67LR and EGCG susceptibility in both human normal cells and myeloma patient samples [7]. Thus, it was postulated that 67LR plays a significant role in tumor progression and studies conducted to define the function of 67LR could provide a new approach to cancer prevention. Most recently, we showed an abrogation of EGCG-induced tumor cell growth inhibition in C57BL/6N mice challenged with 67LR or its downstream signaling molecules, eukaryotic translation elongation factor 1A or myosin phosphatase targeting subunit 1, -knockdowned B16 cells [16]. This is the first report demonstrating the involvement of the EGCG target and its downstream signaling molecules in anti-cancer activity of EGCG *in vivo*. This finding suggests that 67LR and its downstream signaling molecules are “authentic molecular targets”, which determine the efficacy of the cancer-preventive activity of EGCG in a tumor model and have important implications for development and use of EGCG as a cancer-preventive agent.

In recent studies on the anti-cancer action of EGCG, a suggested mechanism of interaction between EGCG and its binding-proteins has been reported [5]. 67LR interacts with many ligands including laminin, cellular pathogenic prion proteins, cytotoxic necrotizing factor 1 from *Escherichia coli*, Sindbis virus, Venezuelan equine encephalitis virus, Dengue virus, and adeno-associated virus, but the underlying interaction mechanism has not been elucidated [8], [9], [17]–[22]. The crystal structure of the partial domain of human 67LR has been reported, and the results suggested that this finding may provide insights into its function and facilitate the design of novel therapeutics targeting 67LR [23]. However, a possible role of the interaction of EGCG with 67LR still remains unclear.

In this study, our aim is to investigate a potential mechanism of the EGCG-67LR interaction responsible for the bioactivities of EGCG. We performed the neutralizing activity assay using various peptides deduced from the extracellular domain corresponding to the 102–295 region of human 67LR. Only a stretch of 10-amino acids, 161 to 170, IPCNNKGAHS, plays a critical role in neutralizing EGCG’s abilities such as the cell-surface binding and its inhibitory effects on cancer cell growth. This result indicates that residues 161-170 contribute to 67LR-mediated cellular response to EGCG stimulation. This is the first evidence indicating the existence of a potential EGCG sensing motif on 67LR.

## Materials and Methods

### Reagents

(–)-Epigallocatechin-3-*O*-gallate (EGCG), catalase, and superoxide dismutase (SOD) were purchased from Sigma Chemical Co. (St. Louis, MO). Synthesized peptides, 9-20-amino acid residue peptides deduced from the extracellular domain corresponding to the 102-295 region of the human 67LR encoding a 295-amino acid were purchased from Thermo Electron GmbH (Ulm, Germany). The anti-human LR polyclonal (F-18) antibody was purchased from Santa Cruz Biotechnology, Inc. (Santa Cruz, CA). The anti-human LR monoclonal antibody (MLuC5) was purchased from NEOMARKERS (Fremont, CA). Anti-β-actin antibody was obtained from Sigma Chemical Co. (St. Louis, MO). Restriction enzymes were purchased from Fermentas International, Inc (Burlington, Canada). The plasmid vectors used were pTARGET™ and pET-30a(+) from Promega Corporation (Madison, WI) and Merck Biosciences (San Diego, CA), respectively. *E. coli* strain JM109 was used as a host for cloning *E. coli* strain BL21 (DE3) (Agilent Technologies, Inc., Santa Clara, CA) was used as a host for the expression of recombinant proteins.

### Cells and Cell Proliferation Test

**Figure 1:**
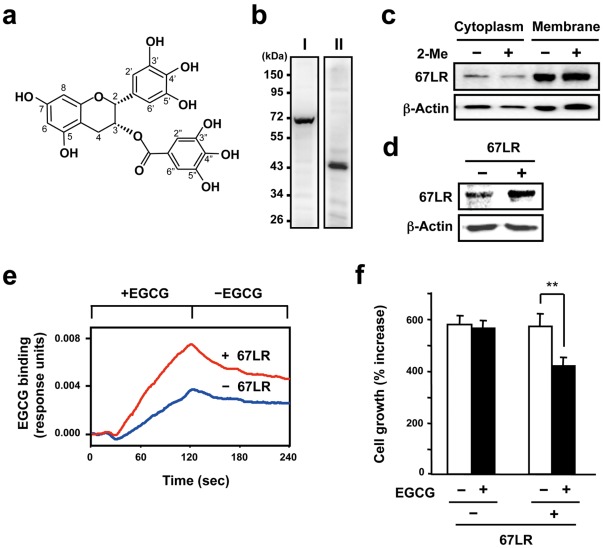
The relationship between the responsiveness of EGCG to the HepG2 cells and 67LR expression. **A**) Chemical structure of green tea polyphenol EGCG. **B**) Western blot analysis of whole cell lysate from HepG2 cells using anti-LR antiserum (I) and anti-LR antibody F-18 (II). **C**) To examine the expression of 67LR on cell membrane in HepG2 cells, both cytosolic and membrane fractions were prepared, and the 67LR were detected by western blot analysis using anti-LR antiserum. This test was performed under reducing (2-Me (+)) or non-reducing (2-Me (−)) conditions. 2-Me indicates 2-mercaptoethanol. The lower panel displays protein levels from the same filter blotted again with the anti-β-actin antibody used as a quantitative loading control. **D**) The cells transfected with either the empty vector (−) or the 67LR gene expression vector (+) were lysed and total cellular protein was subjected to western blot analysis using the cell-surface LR-specific antibody MLuC5. The lower panel displays protein levels from the same filter blotted again with the anti-β-actin antibody used as a quantitative loading control. **E**) Both transfected cells were fixed on the sensor chip. The cell-surface binding of EGCG to immobilized 67LR-overexpressed or control HepG2 cells were measured using a surface plasmon resonance (SPR) biosensor. EGCG was injected at a concentration of 10 µM for the time indicated interval in the figure. **F**) Both types of cells were treated with 1 µM EGCG for 5 days. The results are shown as the relative cell number to untreated control and the data presented are means ± S.D. (n = 3) (Student’s *t*-test, **, *p*<0.01).

To obtain the cells that were highly sensitive to EGCG stimulation, we constructed 67LR-overexpressed cells (Fig. 1). Using standard PCR protocols, cDNA encoding the human 67LR (accession no. NM_002295) was cloned into the expression vector pcDNA3.1(+) (Invitrogen, Carlsbad, CA). Full-length 67LR was amplified by PCR using primers, 5′-CGG TAC CAT GTC CGG AGC CCT TGA TGT CCT GCA AAT G-3′ and 5′-GGC GGC CGC TTA AGA CCA GTC AGT GGT TGC TCC TAC CCA-3′. The 67LR cDNA expression vector (non-tagged type) was transfected into the HepG2 cells, a human hepatocellular carcinoma cell line (ATCC, Rockville, MD), by electroporation. Then, the clone with the neomycin resistance was selected and used for further experiments. The 67LR-overexpressed HepG2 cells, human hepatoma cell line (ATCC), were maintained in Dulbecco’s modified Eagle’s medium (DMEM) (Cosmo Bio, Tokyo, Japan) supplemented with 10% fetal bovine serum (FBS) (Intergen, Purchase, NY), 100 U/ml penicillin G, 100 µg/ml streptomycin, 25 mM HEPES buffer, and 44 mM NaHCO_3_ in a humidified atmosphere with 5% CO_2_ at 37°C. For the cell growth test, HepG2 cells were plated at a density of 2×10^4^ cells/well (24-well culture plate) and allowed to adhere overnight. Each 67LR peptide (1 µM) and EGCG (1 µM) were mixed and incubated at room temperature for 15 min in DMEM supplemented with 200 U/ml catalase and 5 U/ml SOD. Then the mixture was added to the HepG2 cells in DMEM supplemented with 2% FBS, 5 mg/ml BSA, 200 U/ml catalase and 5 U/ml SOD. Following a subsequent incubation of 5 days, the cells in each well were enumerated using a Cell Counter (Sysmex Corporation, Tokyo, Japan). In this study, the cell death was not observed in HepG2 cells treated with or without EGCG.

### Recombinant Proteins of the Extracellular Domain of Human 67LR and its Deletion Mutant

The extracellular domain (amino acid residues 102-295) of human 67LR cDNA fragment, r-hLR_102-295_, with artificial recognition sites of Kpn I and Not I at the 5'- and 3'-ends, respectively, was amplified by PCR from pcDNA3.1(+)-human 67LR plasmid using sense: 5′-C GGT ACC CGC TTC ACT CCT GGA ACC TTC ACT AAC-3′ and anti-sense: 5′-GGC GGC CGC TTA AGA CCA GTC AGT GGT TGC TCC TAC CCA-3′ primers. The resulting product was first ligated to the pTARGET-vector, and transformed into *E. coli* cells JM109. For construction of the deletion mutant (Δ161-170) of r-hLR_102-295_, r-hLR_102-295_Δ161-170, a PCR method was carried out to delete residues 161-170 by using a series of overlapping sense and antisense primer pairs. Sequences of overlapping oligoprimers were as follows: sense primer: 5′-ATT GCC GTG GGT TTA ATG TGG TGG ATG CTG-3′ and anti-sense primer: 5′-TAA ACC CAC GGC AAT GTC CAC ATA GCG CAG-3′. Using these overlapping primers and the extracellular domain-amplifying primers as mentioned above, two DNA fragments with the overlapping end were obtained separately, and these fragments were mixed to hybridize the overlapping ends to each other. The fused DNA fragments were amplified by PCR using the extracellular domain-amplifying primers. The resulting product, r-hLR_102-295_Δ161-170, was ligated to the pTARGET-vector and transformed into *E. coli* cells JM109.

We also attempted to overproduce the full-length human 67LR extracellular domain, r-hLR_102-295_, and the deletion mutant of the 161-170, r-hLR_102-295_Δ161-170. The gene fragments encoding both r-hLR_102-295_ and r-hLR_102-295_Δ161-170 were placed under the control of the T7 promoter on the expression plasmid pET-30a(+) using Kpn I and Not I restriction sites. In these constructs, the gene products were expected to carry a His-tag sequence attached at the N-terminus. Expression in *E. coli* strain BL21 (DE3) was induced overnight with 1 mM IPTG at 20°C as described by the manufacturer. The *E. coli* cells were centrifuged at 5,000 rpm for 10 min at 4°C. The cell pellet was washed several times in Buffer A (50 mM Tris-HCl, pH 8.0, containing 200 mM NaCl) and then sonicated in Buffer A. After centrifugation at 12,000 rpm for 10 min, purification was carried out by using a His-bind resin column (His Trap Chelating HP; GE Healthcare UK Ltd., Buckinghamshire, England), as described by the manufacturer. The proteins were loaded onto the nickel-charged His-bind resin column, previously equilibrated with 15 mL of the binding buffer (20 mM Tris-HCl, pH 7.9, containing 5 mM imidazole and 0.5 M NaCl). After washing with two column volumes of the wash buffer (20 mM Tris-HCl, pH 7.9, containing 60 mM imidazole and 0.5 M NaCl), the adsorbed proteins were eluted with the elution buffer (20 mM Tris-HCl, pH 7.9, containing 1 M imidazole and 0.5 M NaCl). Then, this protein treated with thrombin to remove the His tag, and further purified by gel filtration on a Superose 12 column (10×300 mm; GE Healthcare UK Ltd., Buckinghamshire, England) equilibrated with Buffer A. This purified protein was used for the neutralization activity assay.

### The Cell-surface Binding Analysis of EGCG

**Figure 2:**
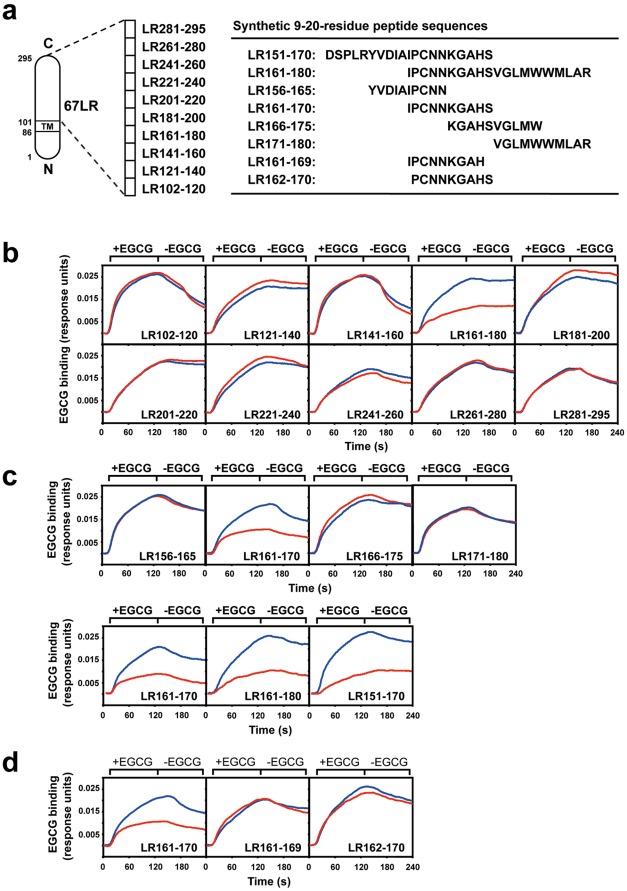
The neutralization of the cell-surface binding of EGCG by peptides deduced from the extracellular domain of 67LR. **A**) Synthetic peptide sequences deduced from the extracellular domain of 67LR. The neutralizing activity of these peptides for the cell-surface binding was assayed. After incubation of EGCG (1 µM) with each peptide (1 µM): (**B**) 20-amino acid residue peptides, (**C**) 10-amino acid residue peptides, or (**D**) 9-amino acid residue peptides (single amino acid deletion form of the N- or C-terminus of the peptide LR 161-170), interactions between these EGCG-peptide mixtures and the 67LR-overexpressed HepG2 cells were measured by a SPR assay. Sensorgrams of the net binding of EGCG, which is the value obtained from subtracting the peptide-binding signal from the total mixture-binding signal, are shown in panels B-D. The results are represented as EGCG alone (blue line) and EGCG plus peptide (red line).

Analysis of the interaction between EGCG and the 67LR-overexpressed HepG2 cells was performed using the surface plasmon resonance (SPR) biosensor SPR670 (Moritex Corp., Tokyo, Japan) as previously reported [6]. The cells were immobilized on the sensor chip and the chip was equilibrated in PBS. EGCG (10 µM) was added at a flow rate of 30 ml/min. The cell-surface binding was measured at 25°C for 2 min followed by dissociation. In this binding analysis, the SPR signal has a characteristic behavior as follows. The elevation of the SPR signal (the value of the changed resonance angle: resonance units) was observed immediately after the injection of the ligands (+EGCG). After the termination of the ligand exposure (-EGCG), the perfusion buffer was changed to the ligand-free running buffer, and the SPR signal was reduced by the dissociation of ligands bound to the surface of the immobilized molecules, and the signal converged to a constant level. For neutralizing experiments (Fig. 2), prior to the EGCG injection to the cells, each 67LR peptide (10 µM) and EGCG (10 µM) were mixed and pre-incubated at room temperature for 15 min in PBS. This mixture was injected to the cells and the binding strength was calculated by subtracting the original binding signal (EGCG +67LR peptide) from the binding signal obtained by injection of each 67LR peptide alone.

### SDS-PAGE and Western Blotting

**Figure 3:**
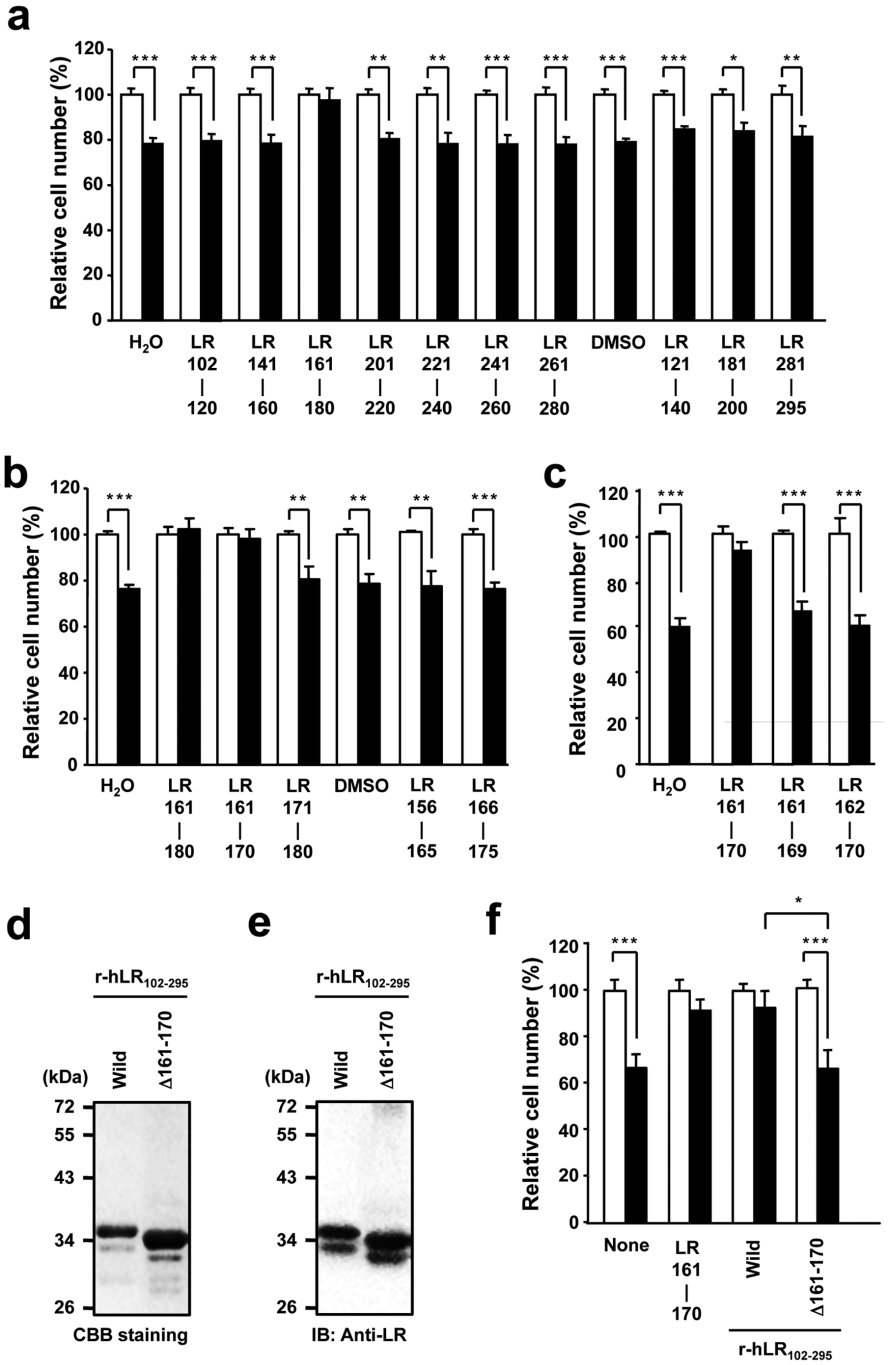
The neutralization of the inhibitory effect of EGCG on cancer cell growth by peptides deduced from the extracellular domain of 67LR. After pre-incubation of EGCG (1 µM) with each peptide (1 µM): (A) 20-amino acid segment peptides, (B) 10-amino acid segment peptides, or (C) 9-amino acid segment peptides (single amino acid deletion form of the N- or C-terminus of the peptide LR 161-170), the 67LR-overexpressed HepG2 cells were treated with these mixtures for 5 days and the cell number was assessed. The results, EGCG plus peptide (closed bar), are shown as the relative cell number to the EGCG-nontreated control (open bar), and the data presented are the means ± S.D. (n = 3) (Student’s *t*-test, *, *p*<0.05, **, *p*<0.01, ***, *p*<0.001). D) SDS-PAGE of recombinant LR (r-hLR_102-295_ and the mutant r-hLR_102-295_Δ161-171 expressed in *E. coli*). The lanes of Wild and Δ161-171 are the wild-type r-hLR_102-295_ and the mutant r-hLR_102-295_Δ161-171, respectively. Molecular mass markers (in kDa) are indicated at the left. E) Western blot analysis of the recombinant LR was performed by using the anti-LR antibody F18. F) The effect of r-hLR_102-295_ and the mutant r-hLR_102-295_Δ161-170 on the cancer cell growth inhibition by EGCG. After incubation of each r-hLR protein or LR161-170 peptide with or without EGCG, HepG2 cells were treated with these mixtures for 5 days and the cell number was assessed. The results are shown as the relative cell number of EGCG-, EGCG plus LR peptide-, or EGCG plus LR protein-treated cells (closed bar) to the EGCG-nontreated control cells (open bar) under each mixture condition (none, LR peptide, or LR protein), and the data presented are the means ± S.D. (n = 3) (Student’s *t*-test, ***, *p*<0.001).

For validating the expression of 67LR in HepG2 cells transfected with or without the 67LR expression vector (Fig. 1), the total cellular level of 67LR expression from whole cell lysate was measured by western blotting. The cells were lysed in cell lysis buffer containing 50 mM Tris-HCl (pH 7.5), 150 mM NaCl, 1% triton-X 100, 1 mM EDTA, 50 mM NaF, 30 mM Na_4_P_2_O_7_, 1 mM phenylmethylsulfonyl fluoride, 2.0 mg/ml aprotinin, and 1 mM pervanadate. Whole cell lysate was incubated at 4°C for 30 min and then centrifuged at 15,000 g for 30 min. The supernatant or purified recombinant LR protein (Fig. 3) was mixed with sodium dodecyl sulfate-polyacrylamide gel electrophoresis (SDS-PAGE) sample buffer. The mixture was loaded onto a 10% SDS-PAGE gel, and electrophoresis was done under reducing conditions (addition of 2-mercaptoethanol). For detection of 67LR expression in the plasma membrane of HepG2 cells (Fig. 1C), we separated cytoplasmic and membrane proteins by using a membrane protein extraction kit (Fermentas, Burlington, Canada), and the proteins were mixed with SDS-PAGE sample buffer. These samples were loaded onto a 10% SDS-PAGE gel, and electrophoresis was done under reducing or non-reducing conditions. Visualization of all proteins on SDS-PAGE gel was performed by Coomassie Brilliant Blue (CBB) staining (Fig. 3). The sample after electrophoresis was then electrotransferred onto a nitrocellulose membrane. The blotted nitrocellulose was probed for 37LRP/67LR using anti-LR antibody (F18 or MLuC5) or anti-LR antiserum, which was obtained from rat immunized with synthesized peptide deduced from residues 161-170 of the human 67LR as previously reported [24]. The secondary antibodies used were HRP-conjugated anti-rabbit IgG or anti-mouse IgM, and detection was done using the ECL kit (GE Healthcare UK Ltd., Buckinghamshire, England).

### Electrospray Ionization Mass Spectroscopy

The peptide solution was prepared by incubating each peptide (5 µM) with or without EGCG (5 µM) in water at room temperature for 15 min. The solution was dissolved in acetic acid (1%)/ethanol (50%) solution, and the dissolved samples were subjected to mass spectrometric analysis. Mass spectra of the complexes were acquired on a LCQ Advantage ion trap mass spectrometer (Thermo Fisher Scientific Inc., MT) fitted with an ion source spray, electrospray ionization (ESI), working in the positive ionization mode. The capillary was typically held at 4.5 kV and 270°C. The dissolved samples were continuously infused into the ESI chamber at a flow rate of 100 µl/min.

## Results

### Construction of the Cells Capable of 67LR-mediated Cellular Response to a Physiologically Relevant EGCG

The 67LR protein is believed to be derived from a smaller precursor, the 37-kDa laminin receptor precursor (37LRP). However, the precise mechanism by which cytoplasmic 37LRP becomes cell-membrane-embedded 67LR is still unclear [25]. In addition, expression patterns of 37LRP and 67LR in cancer cells remain poorly understood. To examine the relationship between 37LRP/67LR and the bioactivity of the major green tea polyphenol EGCG (Fig. 1A), we performed western blot analysis of whole cell lysate from HepG2 cells, a human hepatocellular carcinoma (Fig. 1B). The LR protein corresponding to approximately 67 kDa, 67LR, was observed by using the anti-LR antiserum (I), but the expression of the 37-kDa form (37LRP) was not detected. On the other hand, the anti-LR antibody F18 (II) was able to recognize the 37LRP, but not the 67LR. In HepG2 cells, both types of LR, the 37-kDa and 67-kDa forms, were observed. Previously, we have reported that the level of total cellular 67LR expression is strongly associated with the bioactivities of EGCG [6], [16], [26]–[29], and in particular, the lipid-raft-associated 67LR on the plasma membrane plays an essential role in governing both the localization of 67LR and the cellular responsiveness of EGCG [26]. Therefore, it is important to investigate the localization of 67LR in HepG2 cells to obtain a better understanding of the cell-surface 67LR-mediated bioactivities of EGCG. Here we revealed that the expression level of 67LR in the plasma membrane fraction was much higher than that in the cytoplasmic fraction (Fig. 1C). These results raise the possibility that the plasma membrane 67LR may be responsible for the cell-surface-mediated bioactivity of EGCG in HepG2 cells. Until now, the most common explanations of the process of 67LR are post-translational covalent modification or the formation of very tightly associated homo- or hetero-dimers [30]–[32]. There are two nucleophilic cysteine residues (143 and 163) in the extracellular domain (102-295) of 37LRP/67LR. To examine whether or not a Cys residue-mediated covalent modification, such as disulfide bond formation, is involved in the formation of the cell-surface 67LR in HepG2 cells, we performed western blot analysis of the 67LR in the presence or absence of the reducing agent, 2-mercaptoethanol (2-Me). The amount of 67LR was not affected regardless of the presence or absence of the reducing agent (Fig. 1C). This result suggests that a Cys residue-mediated disulfide bond is not involved in the formation of the cell-membrane 67LR.

To further examine the relationship between the cell-surface 67LR-mediated cellular response and its interaction with EGCG more sensitively, we constructed 67LR-overexpressed cells by stable transfection of a 67LR gene expression vector into HepG2 cells. Western blot analysis of total cellular expression of 67LR using the anti-LR antibody MLuC5, which can recognize the cell-surface LR and inhibit the cell-surface LR-mediated bioactivity of EGCG [6], [26], revealed that the expression of 67LR protein was elevated in 67LR-overexpressed cells (Fig. 1D). To investigate whether an increase in the expression of 67LR affects the interaction of EGCG with the cells, we measured the cell-surface binding of EGCG using a surface plasmon resonance (SPR) biosensor. This technique has been increasingly used for real-time analysis of the binding between solubilized molecules and molecules immobilized on the surface of a biosensor chip without any labeling by changes in the refractive index of a biospecific surface [6], [26], [33]–[35]. SPR analysis revealed that the binding of EGCG to the 67LR-overexpressed cells (+67LR) was higher than that of the cells transfected with the empty vector (-67LR, Fig. 1E). This result and the fact that 67LR is predominantly expressed in the plasma membrane (Fig. 1C) suggest that the increase of EGCG’s binding to the cells may be due to the elevated binding to the cell-surface 67LR. We next investigated whether this binding increase affected the inhibitory effect of EGCG on the proliferation of HepG2 cells at the low concentration of 1 µM, a physiologically relevant concentration [5]. As shown in Fig. 1F, there was no alteration of the cell growth in control cells (-67LR) upon treatment with EGCG, while a significant inhibition of proliferation without inducing cell death was observed in the 67LR-overexpressed cells. These results indicate that the cell-surface 67LR plays an essential role in the anti-cancer action of a physiologically relevant concentration of EGCG. Thus, the 67LR-overexpressed HepG2 cells were used in the subsequent experiments.

### Neutralization Activity of Various Peptides Deduced from the Extracellular Domain of 67LR Against the Cell-surface Binding of EGCG

To identify the EGCG-binding site of the 67LR, we synthesized ten kinds of 20-amino acid residue peptides deduced from the extracellular domain corresponding to the 102-295 region of human 67LR encoding 295-amino acids as shown in Fig. 2A. The neutralizing effects of these peptides on the cell-surface binding of EGCG were investigated using a SPR biosensor (Fig. 2B). EGCG was shown to be able to bind to the 67LR-overexpressed HepG2 cells (blue line). As seen for the case of EGCG plus peptide (red line), the binding strength was clearly reduced by peptide LR161-180, IPCNNKGAHSVGLMWWMLAR, but the other peptides did not reduce the interaction. These observations suggest that EGCG binds to a specific 20-amino acid residue peptide corresponding to the 161-180-residue stretch of the extracellular region of the 67LR.

To further define the potential binding site for EGCG, we conducted the same SPR assay using four kinds of the shorter 10-residue peptides deduced from the 161-180 residue region of the 67LR (Fig. 2C). Only one peptide LR161-170, IPCNNKGAHS, resulted in a reduction of the cell-surface binding of EGCG. We also performed an additional SPR test to clarify whether the 10-amino acid length was required for the neutralizing activity of the peptide LR161-170 (Fig. 2D). The neutralizing activity of LR161-170 was inhibited by a single amino acid deletion of the N- or C-terminus (I^161^ or S^170^) of LR161-170. These data indicate that a stretch of 10-amino acid residues 161 to 170 (IPCNNKGAHS) in the extracellular region of the 67LR is indispensable for neutralization activity against the cell-surface binding of EGCG, and may contribute to the EGCG-67LR interaction.

### Estimation of the Potential Sensing Motif on the Extracellular Domain of 67LR Contributed to the Inhibitory Effect of EGCG on Cancer Cell Proliferation

We next examined the neutralizing activity of the 20-residue peptides (Fig. 3A) for EGCG's ability to inhibit the proliferation of the 67LR-overexpressed HepG2. After pre-incubation of each 67LR peptide (1 µM) and EGCG (1 µM), this mixture was added to the cells once at 0 day and further cultured for 5 days. The results are shown as the relative cell number of EGCG- or EGCG plus LR peptide-treated cells (closed bar) to EGCG-nontreated control cells (open bar) at each mixture condition (none or LR peptide). Addition of the peptide alone did not affect the growth of the cells. Among the 20-amino acid residue peptides tested, the peptide LR161-180 almost completely abolished the anti-proliferative effect of EGCG, while the other peptide did not. Thus, the 20-amino acid residue corresponding to the 161-180-residue region of the extracellular domain of the 67LR may play an important role in the anti-proliferation activity of EGCG.

To narrow the potential binding sequence for EGCG, an additional cell proliferation assay using four kinds of the shorter 10-residue peptides deduced from the 161-180 residues of the 67LR was performed (Fig. 3B). Among the four peptides, only peptide LR161-170 caused a reduction in the anti-proliferation action of EGCG. Furthermore, the neutralizing activity of LR161-170 was prevented by a single amino acid deletion at the N- or C-terminus (I^161^ or S^170^) of LR161-170 (Fig. 3C). These results implicate that a stretch of 10-amino acids from 161 to 170 in the extracellular region of the 67LR is crucial for the neutralizing activity towards the anti-proliferation as well as the cell-surface binding.

To extrapolate these findings to the protein level, we constructed the recombinant protein corresponding to the extracellular domain sequence 102 to 295 of human 67LR, r-hLR_102-295_, and the Δ161-170 deletion mutant, r-hLR_102-295_Δ161-170, in an *E. coli* expression system. Two different molecular weight bands were observed by both SDS-PAGE and western blot analysis of recombinant LR protein r-hLR_102-295_ (Figs. 3D and 3E). The bands of the wild-type r-hLR_102-295_ were approximately 33 and 35 kDa, and the corresponding bands of the mutant r-hLR_102-295_Δ161-170 were approximately 32 and 34 kDa. Because the theoretical size of the 10-amino acid residue fragment (161-170) is 1,040 Da, the difference in size between the wild and mutant types, approximately 1 kDa, is reasonable, indicating successful construction of the LR mutant, r-hLR_102-295_Δ161-170. At least, such a band pattern of both types of r-hLR (Figs. 3Dand 3E) suggests that two species of bands are not intrinsic proteins from *E. coli* but extrinsic recombinant proteins. At present, we can’t exclude the possibility of some sort of difference in modification or folding of recombinant LR protein in *E. coli*. Further study is required for elucidating details of this structural difference. Previously, Vana and Weiss reported that the expression of the mutant LRP102-295::FLAG in mouse neuroblastoma (N2a) and baby hamster kidney (BHK) cells, respectively, resulted in a molecular weight of approximately 33 kDa [36]. Although the expression system, host and vector, were different, the molecular size of this recombinant was very similar to that of the r-hLR_102-295_ expressed by *E. coli*. After pre-incubation of each recombinant LR protein, wild-type r-hLR_102-295_ or mutant r-hLR_102-295_Δ161-170, or LR161-170 peptide with or without EGCG, these mixtures were added to the 67LR-overexpressed HepG2 cells. We evaluated the neutralizing activity of the recombinant proteins towards EGCG’s ability to inhibit the proliferation of HepG2 cells (Fig. 3F). The results are shown as the relative cell number of EGCG-, EGCG plus LR peptide-, or EGCG plus LR protein-treated cells (closed bar) to EGCG-nontreated control cells (open bar) at each mixture condition (none, LR peptide, or LR protein). Treatment with r-hLR_102-295_ protein neutralized the inhibitory effect of EGCG towards cancer cell growth to the same extent as the peptide LR161-170. The deletion of residues 161-170 in r-hLR_102-295_ caused a significant reduction of the neutralizing activity of r-hLR_102-295_. Taken together, these observations provide clear evidence that residues 161-170, IPCNNKGAHS, in the extracellular region of human 67LR are responsible for the 67LR-mediated anti-cancer activity of a physiologically relevant EGCG.

### Characterization of the Functional Motif Responsible for Exerting EGCG’s Activities

**Figure 4:**
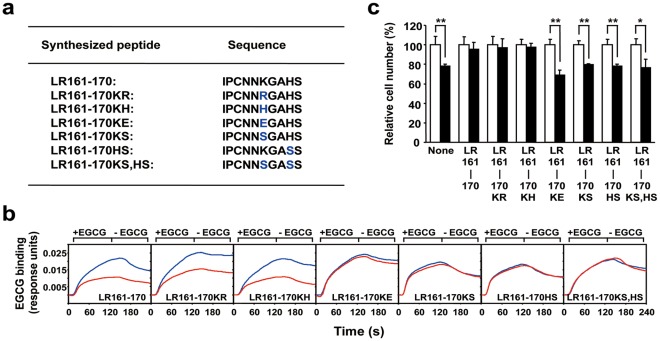
Importance of basic amino acid resides in the EGCG sensing motif for EGCG’s activity. **A**) Basic amino acid replacements of LR161-170. Replaced peptide sequences are shown in the list. **B**) The neutralizing activity of several basic amino acid-replaced LR161-170 segments for the cell-surface binding of EGCG. After incubation of EGCG with each peptide at a molar ratio of 1∶1 in PBS, interactions between these EGCG-peptide mixtures and the cells were measured by a SPR assay. Sensorgrams of net binding of EGCG, which is the value of the subtracted peptide-binding signal from the total mixture-binding signal, are shown. The results are represented as EGCG alone (blue line) and EGCG plus deletion mutant of LR161-170 (red line). **C**) The neutralizing activity of several basic amino acid-replaced LR161-170 segments on the EGCG-induced inhibition of cancer cell growth. After incubation of EGCG with each peptide, the 67LR-overexpressed HepG2 cells were treated with the mixtures for 5 days and the cell number was assessed. The results, EGCG plus peptide (closed bar), are shown as the relative cell number to the EGCG-nontreated control (open bar), and the data presented are the means ± S.D. (n = 3) (Student’s *t*-test, *, *p*<0.05, **, *p*<0.01).

Several lines of evidence regarding the interaction between EGCG and peptides or proteins suggest the importance of the presence of nucleophilic residues [37], [38]. To elucidate the detailed mechanism for the interaction between EGCG and the 161-170 amino acid sequence on 67LR contributing to the neutralization activity, we examined the nucleophilic basic amino acid replacement of LR161-170, as shown in Fig. 4A. Replacement of K^166^ with other basic amino acids R or H did not affect the neutralization activity such as the cell-surface binding or the inhibitory effect on cancer cell growth (Figs. 4B and 4C). On the other hand, replacement of K^166^ with an acidic or neutral amino acid such as E or S showed an inhibition of both neutralization activities of the peptide (Figs. 4B and 4C). These results suggest that a basic amino acid at position 166 of LR161-170 is essential for exerting the neutralization activities of the peptide.

In addition to these findings, we also investigated the involvement of another basic amino acid H^169^ in both neutralizing activities. As shown in Figs. 4B and 4C, both neutralization activities were inhibited by the replacement of H^169^ with S. Replacement of both K166 and H169 to S also caused the inhibition of both neutralization activities. These facts indicate that the existence of both K^166^ and H^169^ is indispensable for neutralizing the cell-surface binding and the inhibitory effect on cancer cell growth. Therefore, these two basic amino acids, K^166^ and H^169^, on the extracellular domain of human 67LR may contribute to the EGCG-67LR interaction mediating functions of EGCG.

**Figure 5:**
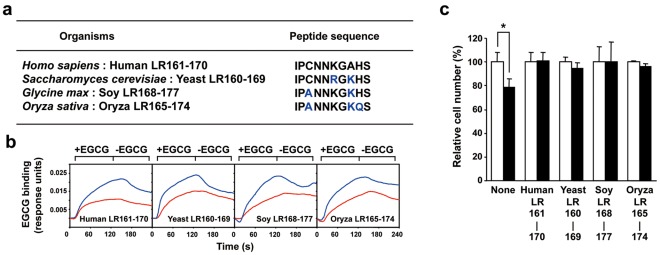
A possible role of LR161-170 motif derived from other organisms for exerting of EGCG’s activities. **A**) LR161-170 motif of other organisms. **B**) The neutralizing activity of LR161-170 derived from other organisms for the cell-surface binding of EGCG. After incubation of EGCG with each peptide at a molar ratio of 1∶1 in PBS, interactions between these EGCG-peptide mixtures and the 67LR-overexpressed HepG2 cells were measured by a SPR assay. Sensorgrams of the net binding of EGCG, which is the value of the subtracted peptide-binding signal from the total mixture-binding signal, are shown. The results are represented as EGCG alone (blue line) and EGCG plus deletion mutant of LR161-170 (red line). **C**) The neutralizing activity of LR161-170 derived from other organisms on the EGCG-induced inhibition of cancer cell growth. After incubation of EGCG with each peptide, HepG2 cells were treated with the mixtures for 5 days and the cell number was assessed. The results, EGCG plus peptide (closed bar), are shown as the relative cell number to the EGCG-nontreated control (open bar), and the data presented are the means ± S.D. (n = 3) (Student’s *t*-test, *, *p*<0.05).

In this study, we described a possible role of EGCG sensing motif in human 67LR. However, it is unclear whether this role is applicable to other organisms. The neutralization activity assay using peptides derived from other organisms corresponding to the human LR161-170 was performed. Although 4 species each have a different peptide sequence in this region (Fig. 5A), both neutralization activities were observed in all tested organisms (Figs. 5B and 5C). These results implicate the possibility that 67LR may be able to mediate the biological activity of EGCG in humans as well as the other three organisms. Recently, EGCG has been shown to form a covalent interaction with a nucleophilic cysteine residue in a peptide or protein, suggesting that this interaction may contribute to the biological activity, including the toxicology and anti-tumor mechanism of EGCG *in vivo* [37], [38]. However, our findings that the replacement at position 163 from C to A did not affect the neutralization activities indicated that C^163^ was not responsible for these effects. The results of Figs. 4 and 5 show the essential role of the nucleophilic basic amino acid, K^166^, in human LR161-170 (IPCNNKGAHS). We also described the importance of another basic acid at position 169 (Fig. 4). Furthermore, the ability of the LR peptide was sustained even if the position of basic amino acid changed from 169 to 168, as shown by the result of oriza LR165-174 (IPANNKGKQS, Fig. 5). Therefore, two basic amino acids within the region 166-169 in the human LR as well as the 10-amino acid segment appear to be at least indispensable for unraveling the structural properties of 67LR.

**Figure 6:**
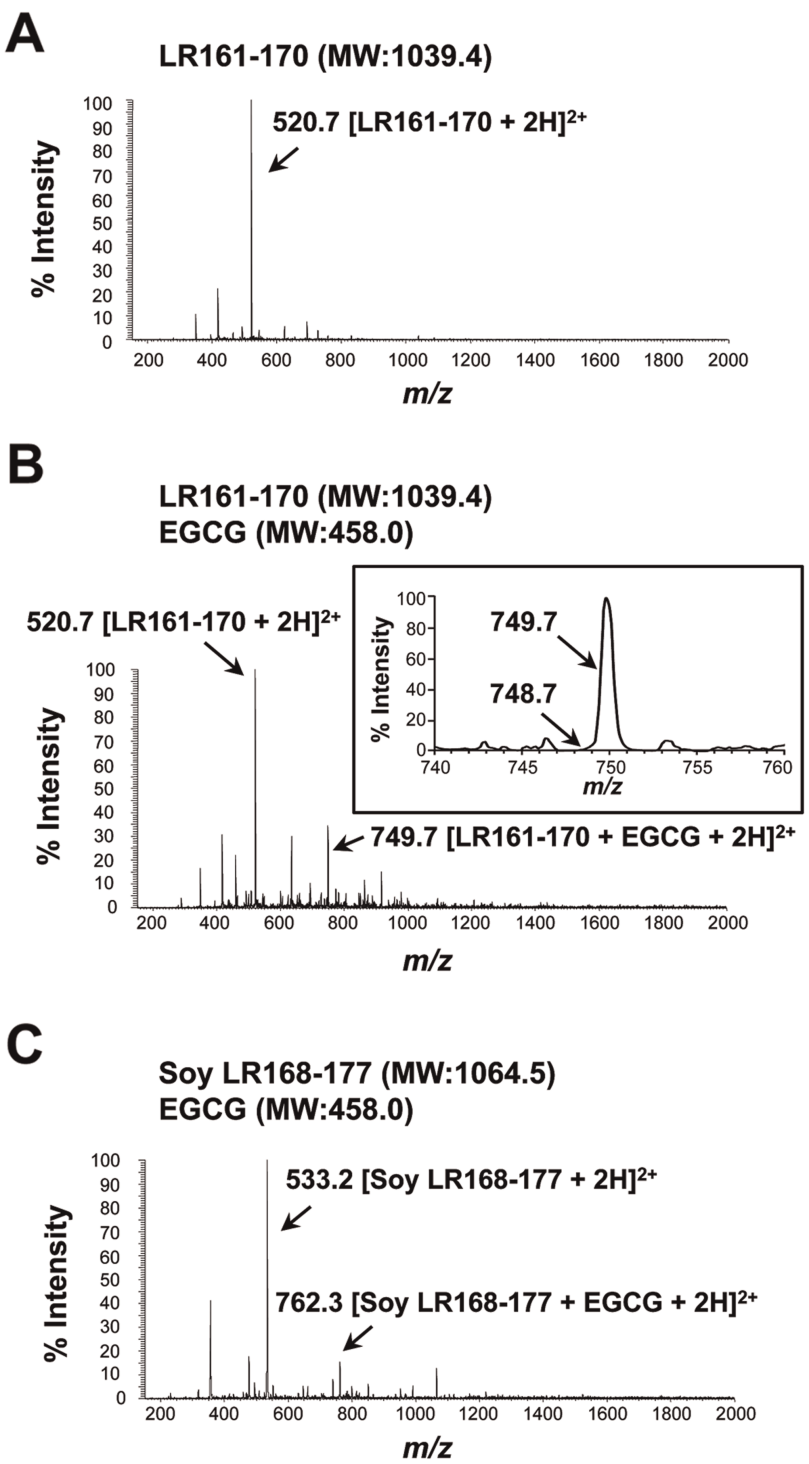
The nature of the EGCG-LR peptide interactions. Electrospray ionization mass spectrum of peptide (**A**) or peptide-EGCG mixture (**B, C**). The peptide solution was prepared by incubating each peptide (5 µM) with or without EGCG (5 µM) in water at room temperature for 15 min. Solutions of the mixture were analyzed in the positive ion mode. The arrows in the figure show the observed ion peaks. **A**) The Cys residue-containing peptide human LR161-170 and (**B**) its mixture with EGCG. **B**) An inset is the figure zooming in the relevant peak to see the difference between non-covalent (*m/z* 749.7 [LR161-170+EGCG+2H]^2+^) and covalent binding (*m/z* 748.7 [(LR161-170+EGCG-2H)+2H]^2+^). **C**) Mass spectrum of the mixture of the Cys residue-lacking peptide soy LR168-177 with EGCG. [LR161-170+2H]^2+^ or [Soy LR168-177+2H]^2+^ represents the doubly protonated form of each peptide. [LR161-170+EGCG+2H]^2+^ or [Soy LR168-177+EGCG+2H]^2+^ represents the peptide ion with added EGCG (EGCG-peptide complexes).

Although we described the characterization of the functional motif responsible for exerting EGCG’s activities, the precise nature of EGCG-LR peptide interaction, including whether it is a covalent or non-covalent complex or forms a monomer or dimer, still remained unclear. To directly address this issue, we performed mass spectrometric analysis, which can identify peptide-peptide or low molecular weight compound-peptide interactions [37], [39] (Fig. 6). In the Cys residue-containing and neutralizing activity-bearing peptide LR161-170, the mass spectrum revealed protonated ion peak at *m/z* 520.7, corresponding to the doubly protonated monomer peptide peak [LR161-170+2H]^2+^ (Fig. 6A). In contrast, a dimer peptide peak, which was caused by formation of a disulfide bond between two Cys^163^ residues, was not observed at *m/z* 1039.4 [Dimer (2LR161-170-2H)+2H]^2+^. Although further study is necessary, the results of the Cys residue-targeted analysis, both LR peptide (Fig. 6) and 67LR protein (Fig. 1C), provide the possibility that the 67LR is not due to the homo-dimerization of 37LRP directly mediated through the Cys^163^ residue within residues 161-170. We also analyzed the mixture of LR161-170 peptide with EGCG (Fig. 6B). The mass spectrum showed an ion peak at *m/z* 749.7 corresponding to the non-covalent ion peak of chemically non-altered EGCG (intact form)-monomer peptide complex [LR161-170+EGCG+2H]^2+^. However, the ion peak of a EGCG-dimer peptide complex was not detected at *m/z* 1268.5, corresponding to [Dimer (2LR161-170-2H)+EGCG+2H]^2+^. These data suggest that mixing EGCG with the peptide does not induce the chemical alteration of EGCG, and instead a non-covalent complex, based on intact EGCG-monomer LR peptide interactions, may play an essential role in the EGCG-67LR interaction and bioactivity of EGCG. Although previous studies have reported the covalent interaction between EGCG and a certain Cys residue-containing peptide, EGCG-2′-Cys-peptide [37], a covalent complex peak [(LR161-170+EGCG-2H)+2H]^2+^ was not observed at *m/z* 748.7 (Fig. 6B, inset). Therefore, these mass spectral data indicated that EGCG could not form a covalent complex with the monomer LR161-170 peptide. As shown in Fig. 5, soy LR168-177 peptide corresponding to the human LR161-170 is lacking a Cys residue but still has neutralizing activity. Mass spectrometric analysis revealed an ion peak of chemically non-altered EGCG (intact form)-monomer peptide complex [Soy LR168-177+EGCG+2H]^2+^ at *m/z* 762.3 (Fig. 6C). This data also support the idea that the Cys residue is not involved in the EGCG-LR peptide interaction, and thus this nucleophilic residue is not responsible for the neutralizing activity of the peptide. Taken together, the binding nature of EGCG to residues 161-170 of 67LR may play an important role in the cell-surface 67LR-mediated bioactivity of EGCG. The proposed role of the EGCG-functional motif may be further defined as more structural experimental evidence is obtained.

## Discussion

The evolutionary analysis of 67LR has suggested that the acquisition of the laminin-binding capability is linked to the palindromic sequence LMWWML, corresponding to residues 173–178 [11]. Intriguingly, this segment is proximate to the EGCG-functional domain, residues 161-170. We have previously reported that a laminin retreatment partially inhibited the binding of EGCG to the 67LR and treatment with the cell-surface LR-specific antibody MLuC5, capable of inhibiting laminin binding to 67LR, prevented EGCG’s abilities such as its cell-surface binding and inhibition of cancer cell growth [6]. Thus, the position proximity between the EGCG- and laminin-binding motifs may provide a possible explanation, steric hindrance caused by laminin or antibody, for the inhibition of the functions of EGCG. In spite of the proximity between both motifs, laminin did not show an inhibitory effect on cancer cell growth in a similar fashion as EGCG [16]. These facts imply that our proposed EGCG sensing motif may not be easily regulated by a proximal region-specific ligand. From the crystal structure of 67LR, residues 161-180 have been shown to comprise the linker between β-sheet strand 7 (β7) and α helix E (αE) [23]. The only portion of this sequence that was solvent-accessible was suggested to be residues 165-169 in the β7-αE linker. It was speculated that such residues might be exposed by a conformational change of the C-terminal tail of the receptor. Although these observations may provide a possible rationale for the accessibility of EGCG to the 161-170 region of 67LR, further structural studies are required to elucidate the details of the EGCG-67LR interaction.

The multifunctional 67LR is known to have a role as a receptor for viruses such as Sindbis virus, Dengue virus, adeno-associated virus, and Venezuelan equine encephalitis virus as well as prion protein [9], [18]–[21], [40]. A hallmark in prion diseases is the conformational transition of the cellular prion protein (PrP^c^) into a pathogenic conformation, designated scrapie prion protein PrP^Sc^, which is the essential constituent of infectious prions. Recently, EGCG has been reported to interfere with the stress-protective activity of PrP^c^ and the formation of PrP^Sc^ in scrapie-infected N2a cells [41]. Interestingly, the 37-kDa/67-kDa laminin receptor has found to be involved in prion functions, and a yeast two-hybrid system showed that the direct PrP-binding site was located between aa 161-179 of the 37-kDa laminin receptor [32]. Thus, our identification of a putative EGCG-binding sequence has also implications for elucidating the potential molecular mechanisms into how EGCG might ameliorate prion diseases.

Protein interactions with EGCG have been proposed to be involved in various bioactivities. Many proteins have been identified as EGCG-binding targets mediating anti-cancer action [5]. Nonetheless, in the most cases, the precise mechanism by which the binding of EGCG to proteins regulates protein function remains to be elucidated. In contrast, the present study is the first evidence providing structural information about the functional domain of 67LR responsible for mediating the anti-cancer action of a physiologically relevant EGCG (1 µM). Previously, the 67LR has been shown to be responsible for anti-allergic effects of EGCG in basophils [26], [34]. In preadipocytes, EGCG has been reported to mediate anti-insulin signaling via the 67LR pathway [42]. Furthermore, protection of dystrophic muscle cells with EGCG correlated with improved glutathione balance and increased expression of 67LR [43]. Most currently, we found that a physiologically relevant EGCG downregulates Toll-like receptor-2/4 signaling through 67LR in macrophages [28], [29]. In addition to these 67LR-mediated cellular responses, chemical modifications of EGCG based on the structural information for its binding molecule have received considerable attention as potential strategies for enhancing biological activities of EGCG [44], [45]. Thus, the structural characterization of 67LR will contribute to a better understanding of how EGCG interacts with 67LR and should help in the design of therapeutics that can mimic EGCG-67LR interactions in preclinical and clinical settings of cancer as well as immune responses, muscle and adipocyte functions.
